# Modelling the impact of relaxing COVID‐19 control measures during a period of low viral transmission

**DOI:** 10.5694/mja2.50845

**Published:** 2020-11-18

**Authors:** Nick Scott, Anna Palmer, Dominic Delport, Romesh Abeysuriya, Robyn M Stuart, Cliff C Kerr, Dina Mistry, Daniel J Klein, Rachel Sacks‐Davis, Katie Heath, Samuel W Hainsworth, Alisa Pedrana, Mark Stoove, David Wilson, Margaret E Hellard

**Affiliations:** ^1^ Burnet Institute Melbourne VIC; ^2^ University of Copenhagen Copenhagen Denmark; ^3^ Institute for Disease Modeling Bellevue WA United States of America

**Keywords:** Theoretical models, COVID‐19, Public policy, Infectious diseases, Respiratory tract infections

## Abstract

**Objectives:**

To assess the risks associated with relaxing coronavirus disease 2019 (COVID‐19)‐related physical distancing restrictions and lockdown policies during a period of low viral transmission.

**Design:**

Network‐based viral transmission risks in households, schools, workplaces, and a variety of community spaces and activities were simulated in an agent‐based model, Covasim.

**Setting:**

The model was calibrated for a baseline scenario reflecting the epidemiological and policy environment in Victoria during March–May 2020, a period of low community viral transmission.

**Intervention:**

Policy changes for easing COVID‐19‐related restrictions from May 2020 were simulated in the context of interventions that included testing, contact tracing (including with a smartphone app), and quarantine.

**Main outcome measure:**

Increase in detected COVID‐19 cases following relaxation of restrictions.

**Results:**

Policy changes that facilitate contact of individuals with large numbers of unknown people (eg, opening bars, increased public transport use) were associated with the greatest risk of COVID‐19 case numbers increasing; changes leading to smaller, structured gatherings with known contacts (eg, small social gatherings, opening schools) were associated with lower risks. In our model, the rise in case numbers following some policy changes was notable only two months after their implementation.

**Conclusions:**

Removing several COVID‐19‐related restrictions within a short period of time should be undertaken with care, as the consequences may not be apparent for more than two months. Our findings support continuation of work from home policies (to reduce public transport use) and strategies that mitigate the risk associated with re‐opening of social venues.

**The known**: On 8 May, the Australian government released a framework for relaxing COVID‐19‐related restrictions.

**The new**: Our simulation model indicated that more than two months could elapse after some policy changes before COVID‐19 case numbers increased markedly. Large, random gatherings entail the greatest risk of a rise in case numbers, while the risk associated with smaller gatherings of people known to each other is lower.

**The implications**: COVID‐19‐related restrictions should be lifted sequentially and gradually. To minimise public transport use, working from home should continue. Physical distancing restrictions are still required to mitigate the risks of opening pubs and bars.

In March 2020, the Australian government introduced mandatory quarantine for travellers returning from overseas and policies that facilitated physical distancing, including closing pubs, bars, entertainment venues, and places of worship, restricting restaurants and cafes to takeaway trade, and limiting public gatherings to two people.^1^ Over two months, these measures successfully disrupted the spread of coronavirus disease 2019 (COVID‐19); nationally, fewer than 55 cases per day were reported between 12 April and 8 May, after a peak of 469 new cases on 28 March.^2^, ^3^ On 8 May, the federal government released a framework^4^ of policy options for re‐opening social and commercial sectors that allowed states and territories to adopt different local timetables. Public health measures also included increased testing capacity and the contact tracing smartphone app COVIDSafe.^4^

Victoria is the second most populous Australian state (6.7 million people; about 26% of the national total).^5^ Until the end of May, the trajectory of the COVID‐19 epidemic in Victoria was similar to that for Australia as a whole; daily numbers of new diagnoses increased during March (peak, 111 cases on 29 March), then rapidly declined as restrictions were imposed. By 15 May (when we undertook our analysis), 1554 COVID‐19 cases had been confirmed in Victoria, the vast majority among quarantined returned travellers.^2^ As community transmission was low, restrictions were relaxed to allow small social gatherings (13 May), cafes and restaurants to open with physical distancing (1 June), and community sports to resume (22 June). In late June/early July, Victoria experienced a resurgence in infections; 12 674 cases were detected between 14 June and 9 August, with a second peak of 695 new cases on 5 August.^2^ The Victorian experience illustrates that the sequence and timing of relaxing restrictions must be carefully considered to avoid compromising their overall effectiveness. Epidemic modelling can provide insights into the likely impact of relaxing individual control measures.

Epidemic models can be broadly classified as population‐ or individual‐level models. Population‐level models divide a population into a small number of discrete categories based on risk and assume homogeneous mixing and transmission risk within each category. Individual‐level models, using a set of autonomous agents to represent a population, allow more detailed simulation of individual‐level characteristics and human behaviour.^6^ The risk of contracting COVID‐19 is highly heterogeneous and is determined by the contact networks of individuals, which in turn depend on age, household structure, and participation in social and community activities. The impact of interventions for slowing the spread of COVID‐19, including contact tracing and quarantine, are also highly dependent on contact networks and therefore most effectively analysed in individual‐level models.

Modelling of the impact in Australia of the policy changes proposed by the COVIDSafe Australia framework has not been published. Population‐level models^7^, ^8^, ^9^, ^10^ were used to support introducing physical distancing policies in Australia, and agent‐based models are increasingly used to simulate the impact of social distancing measures on COVID‐19 spread in Australia and overseas.^11^, ^12^, ^13^, ^14^, ^15^, ^16^, ^17^, ^18^ However, these models have considered only the implementation of contact tracing, quarantine, or social distancing policies, and not the easing of these measures.

We therefore employed an agent‐based model, Covasim,^19^ to assess the risks associated with relaxing physical distancing and lockdown policies in Victoria during a period of low viral transmission (March–May 2020).

## Methods

### Model overview

The Covasim model has been described in detail elsewhere,^19^ and reports on its application to a number of high transmission settings are available.^20^ In brief, each person in the model is characterised by a set of demographic, disease, and intervention status variables. Demographic variables include age (one‐year brackets); uniquely identified household, school (for people aged 5–18 years) and work (for people aged 18–65 years) contacts; and mean number of daily contacts in a range of community networks and settings (Supporting Information, A). Disease variables include infection status (susceptible, exposed, recovered or dead), viral load (time‐varying), age‐specific susceptibility, and age‐specific probabilities of being symptomatic, disease severity level (mild, severe, critical), and mortality. Person‐level intervention status variables include diagnostic status (untested, tested and waiting for results, tested and received results) and quarantine status (yes, no).

Viral transmission is modelled as occurring when a susceptible individual is in contact with an infectious person in one of their contact networks. The daily probability of transmission per contact with an infected person (transmissibility) is calibrated to match reported epidemic dynamics, and is weighted according to whether the infectious person has symptoms, as well as the type and setting of the contact (eg, transmission is more likely through household than community contacts).

Further model details are reported in the Supporting Information, section A. Model age and network structure are described in the Supporting Information, section B, disease parameters in section C, behavioural and network parameters in section D, and policy changes included in the model in section E.

### Baseline scenario and calibration

A baseline scenario was run for the period 1 March – 30 April, which included the Victorian policy changes during this period (Supporting Information, F). The overall probability of transmission per contact was calibrated so that the model projections matched the reported diagnosis and mortality data.

### Scenario set 1: policy relaxations

We simulated several scenarios with single restrictions lifted from 15 May (the date of analysis): opening pubs and bars; allowing large events; opening cafes and restaurants; allowing community sports; allowing small social gatherings; opening entertainment venues, such as cinemas, theatres; removing work from home directives; and opening schools. The parameter and network configuration changes associated with relaxing each restriction are described in the Supporting Information, section D. For each scenario, new infections were introduced for modelling purposes (five infections on 15 May), to re‐start the epidemic and test the robustness of the new policy configuration to fresh outbreaks.

### Scenario set 2: contact tracing smartphone app

We estimated the threshold population‐level coverage with a contact tracing app (COVIDSafe) needed to mitigate the risks of relaxing different policies that we had found in scenario set 1 to entail the greatest risks: re‐opening pubs and bars, and removing work from home directives. We simulated scenarios with population‐level coverage of the contact tracing app of 0–50%.

### Scenario set 3: physical distancing policies in social venues

We estimated how effective additional policy options — the 4 m^2^ rule, limits on customer numbers, restricting venues to providing outside service — would need to be to reduce the risks associated with the opening of cafes, restaurants, pubs and bars. Opening pubs and bars was selected as an example, as we had found it to be associated with the greatest risk; we simulated scenarios in which transmissibility in pubs and bars was reduced by physical distancing policies by 0–50%.

### Scenario set 4: collection of patron identification records by venues

We estimated the threshold proportion of venue contacts (pubs, bars, cafes, restaurants) that would need to be reliably traceable for a policy of venues collecting mandatory patron identification records to mitigate the risks associated with opening these venues. We simulated scenarios in which pubs and bars were opened and could trace 40–80% of contacts in case of a transmission event.

### Ethics approval

Ethics approval was not required for this study as we analysed publicly available data.

## Results

### Model calibration

The fit of the baseline model scenario was acceptable; it included the initial increase in cases observed and the subsequent decline following implementation of specific policies (Box 1).

Box 1 **Model calibration and baseline projection for the initial epidemic wave in Victoria**

**Figure:**
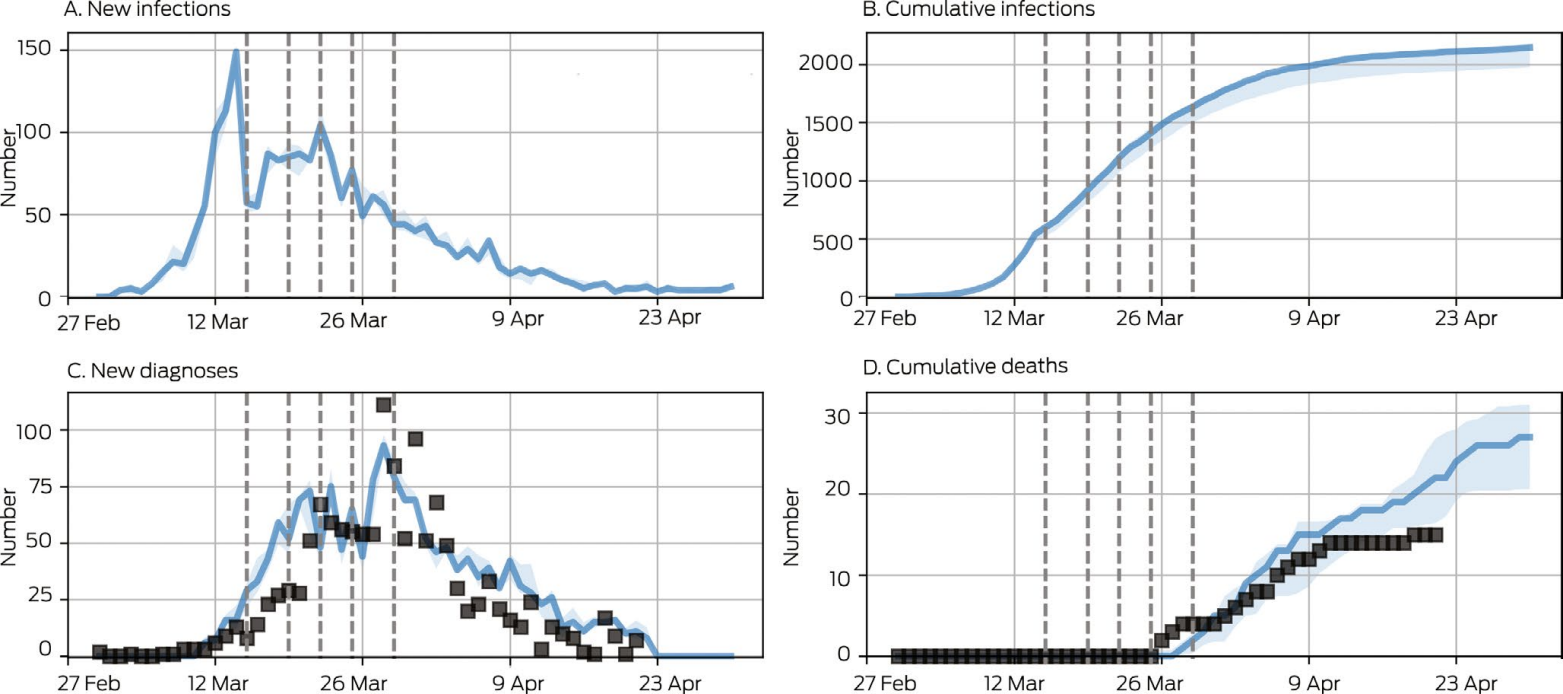
The probability of transmission per contact was varied such that the model matched the observed number of diagnoses and deaths over time. Baseline projections (blue) included policy changes on 19, 21, 22 and 29 March (dashed vertical lines; Supporting Information, F). Black squares represent data; blue lines and shaded areas are model projections (median and interquartile range for 100 simulations). Data were only available for new diagnoses, whereas the model also projects new infections. We estimated that by 30 April about 2000 people were COVID‐19‐positive, of whom about 1600 (80%) had been diagnosed. Most undiagnosed cases were asymptomatic infected persons.

### Scenario set 1: policy relaxations

The greatest risk of renewed increase in case numbers was associated with policies that facilitate random, single‐occasion mixing in the community, or situations in which individuals have large numbers of contacts, particularly with unknown people. These situations include opening pubs and bars (without additional restrictions), removing work from home directives (which increases public transport and work interactions), and allowing large events (concerts, sports events, protests). The least risk was associated with policies that facilitate smaller numbers of contacts, or repeated contacts with the same known people (eg, social gatherings of fewer than ten people). With some policy changes, the time before new infections rapidly increased could be greater than two months; for example, following the opening of cafes and restaurants or entertainment venues (Box 2).

Box 2 **Projected cumulative and new COVID‐19 infections after lifting of specific social restrictions**

**Figure:**
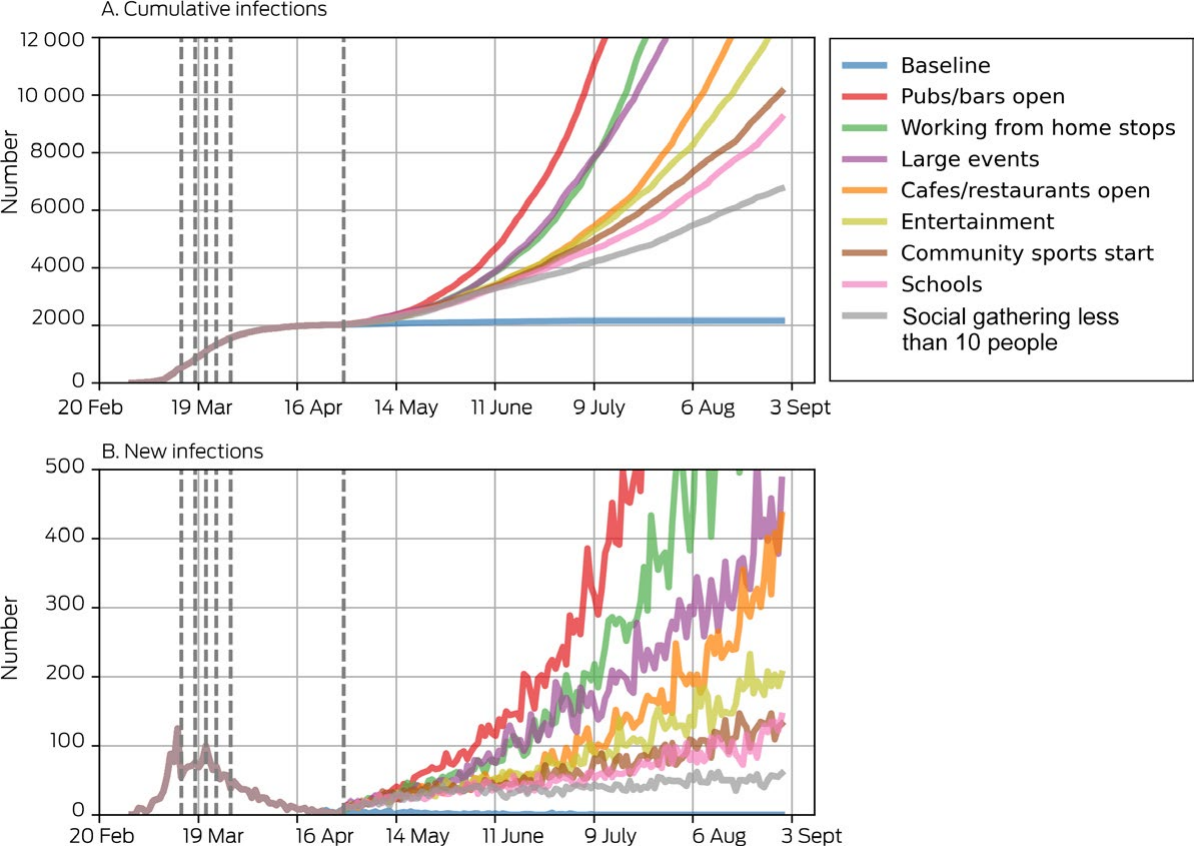
Dashed vertical lines indicate the dates of policy changes. In these projections, venues are modelled as opening without additional physical distancing restrictions (Supporting Information, E), and population‐level coverage of the contact tracing smartphone app was set to 5% (estimated national coverage at 1 May, when the policy changes were simulated, based on about 3 million downloads and 50% appropriate use^21^).

### Scenario set 2: contact tracing smartphone app

Contact tracing app coverage would need to exceed 30% to markedly mitigate population‐level transmission risk after opening pubs and bars (Box 3) or removing work from home directives (Supporting Information, figure 7).

Box 3 **Projected cumulative and new COVID‐19 infections after re‐opening of pubs and bars, by population level of contact tracing smartphone app coverage**

**Figure:**
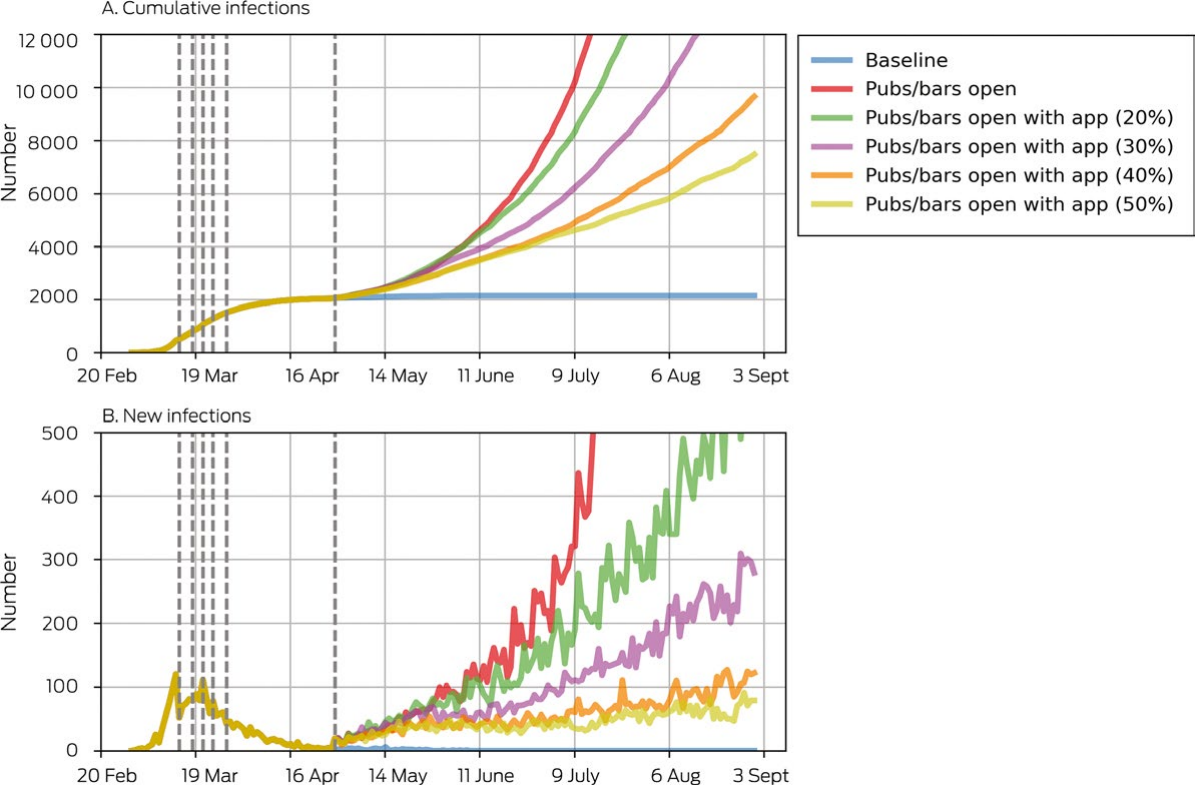
Dashed vertical lines show the dates of policy changes (Supporting Information, F).

### Scenario sets 3 and 4: mitigation strategies in venues

Opening pubs and bars (without additional restrictions) was the policy associated with the greatest increase in new infections. However, the risks could be noticeably reduced if physical distancing in venues depressed transmissibility by at least 40%, with (Supporting Information, figure 8) or without contact tracing app coverage of 25% (Box 4). Alternatively, recording the identification of patrons attending pubs and bars would be effective at the population level if it enabled at least 60% of contacts at these settings to be reliably traced (Supporting Information, figure 9).

Box 4 **Projected cumulative and new COVID‐19 infections after re‐opening of pubs and bars, by reduction of virus transmission with physical distancing measures**

**Figure:**
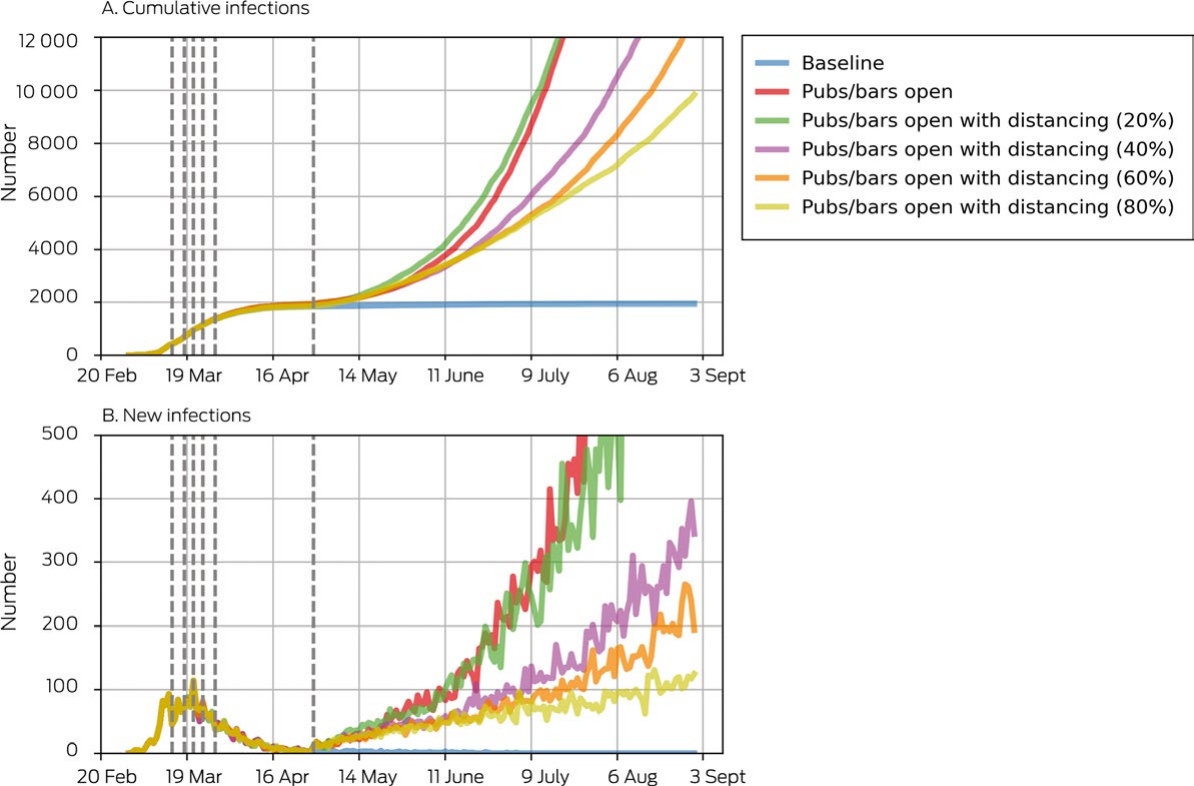
Dashed vertical lines show the dates of policy changes (Supporting Information, F). Population‐level coverage of the contact tracing smartphone app was set to 5% (estimated national coverage at 1 May, when the policy changes were simulated, based on about 3 million downloads and 50% appropriate use^21^).

## Discussion

We simulated, in an agent‐based model, relaxing specific COVID‐19 containment restrictions in a low transmission setting. We found that changes which increase the number of contacts between people unknown to each other — opening pubs and bars, removing work from home directives (leading to greater public transport use), permitting larger public events — were associated with the greatest risks, while changes leading to smaller increases in contacts and with people known to each other (small social gatherings of fewer than ten people) were associated with lower risks. Our model indicates that increases in new infections following a change in policy may be first evident more than two months later. Caution should therefore be taken when sequentially easing multiple restrictions within a short time period (two months), as the consequences of restriction changes may not be immediately apparent. Our findings have implications for other low community transmission settings when governments are planning to remove restrictions after relatively successful early containment.

Despite social and economic pressures to expedite the return to normal conditions, our findings indicate that restraint is needed, even in low transmission settings. In our model, contact tracing is effective for identifying known contacts (Supporting Information, table 7), but transmission through unknown community contacts is possible. For some policy configurations, chains of transmission through unknown contacts may initially account only for a minority of new cases, but can subsequently pose an increasing cumulative risk for epidemic expansion. It is therefore essential that testing services are readily accessible and provide rapid results, complementing contact tracing programs, to ensure timely detection of community transmission through unknown sources.

The greatest risks of a resurgence in case numbers were associated with policy changes that facilitated large contact networks characterised by single‐occasion mixing with unknown people in the community (public transport, pubs and bars, sports events). In particular, our findings supported the Victorian decision to extend working from home recommendations until at least July 2020, to minimise the use of public transport.^1^ Further modelling could assess whether staggered work starting times or increased ventilation and cleaning could mitigate the risks associated with increased public transport use.

Lower risks were associated with policy changes that led to smaller increases in contact numbers for individuals, or which involved organised contact networks of people known to each other who could be more easily traced (eg, social gatherings of fewer than ten people). With these network configurations, population connectivity remains restricted, limiting the potential for more widespread viral transmission. In addition, the probability of timely tracing of known contacts is greater if transmission occurs. However, even for networks of known contacts, the risk of a new rise in case numbers increases with network size.

We found that population coverage with a contact tracing smartphone app (COVIDSafe) would need to exceed 30% to mitigate the risks associated with most policy relaxations. The effectiveness of the app relies on infected and susceptible persons each having compatible phones, downloading the app, and using it correctly. Between its introduction on 26 April and the end of May, there had been an estimated 6 million downloads of the COVIDSafe nationally^21^ (a number corresponding to 24% of the national population); however, no official download number has been published. That is, if each person who downloaded the app did so only once and then used it perfectly, at most an additional 6% (24% × 24%) of contacts could be reliably traced. The app is therefore unlikely to be effective in mitigating risk at this level of coverage.

We estimated that physical distancing strategies that reduce viral transmission in venues by at least 40% are needed to mitigate the risks of opening pubs and bars (the policy change associated with the greatest risk). Our model thereby provides a useful target for interventions including a combination of hygiene measures, physical distancing, and limits on patron numbers. The model also identified that venues keeping records of patrons could be effective if it enabled at least 60% of contacts to be traced (Supporting Information, figure 9). That is, mandatory identification must be more stringent than smartphone app use to be as effective, as the app can assist tracing of multiple generations of transmission, not just those in the source setting.

We found that opening schools was among the lowest risk options, predominantly because school contacts are known, making contact tracing effective in this environment, and because school contact networks (ie, classrooms) did not vary with time for the duration of the simulations, leading to clustered infections rather than broad population spread in the event of an outbreak. In our model, people under 20 years of age were also assumed to be less susceptible to infection than those over 20 (relative susceptibility: 0–9 years, 0.34; 10–19 years, 0.67; Supporting Information, table 2). Further, the probability of people under 20 years of age being symptomatic is lower than for people over 20 (Supporting Information, table 2), and transmission by people with asymptomatic infections was lower in our model. However, the findings of a sensitivity analysis in which susceptibility was equal for all age groups were similar (Supporting Information, figure 10).

### Limitations

Our model attributes only basic properties to individuals: age, household structure, and participation in different contact networks. Consequently, it does not account for demographic and health characteristics such as socio‐economic status, comorbid conditions, and health‐related risk factors (eg, smoking) that may influence transmission risk, testing rates, quarantine adherence, and disease outcomes. The impact of policy changes on different community and geographic population subsets could be assessed in further modelling studies.

The reliability of data on disease parameters used in our models, such as duration of asymptomatic and infectious periods and age‐specific susceptibility, transmissibility and disease severity, will be influenced by differences between surveillance systems in the source countries. Our model could be updated as new information becomes available.

Contact networks are the most important determining factor for viral transmission, but few studies that could provide the parameters needed to model them have been published. The modified Delphi process we employed (Supporting Information, D) was subject to the biases of a non‐randomly selected expert panel, and the wide variation in estimates suggests a high degree of uncertainty about contact network parameters. Nevertheless, it is important to consider contact networks and the impact of policy changes upon them. For example, no published study has quantitatively compared viral transmissibility for public transport and household contacts. However, excluding public transport contacts or assuming that they were equivalent to household contacts would be making a greater unsupported assumption than our Delphi process when estimating the relative transmission risks of public transport and household contacts. Similarly, if people are instructed to work from home, lower transmission associated with public transport would be expected. The degree of reduction is unclear, but excluding this factor would be equivalent to assuming that there was no change. It is critical that these parameters be continually revised as new evidence becomes available.

### Conclusions

In settings of low community viral transmission, care should be taken to avoid changing multiple control policies within a short period, as the consequences for case numbers may not be evident for more than two months. Governments should be particularly wary of lifting restrictions that facilitate larger numbers of contacts between people who do not know each other, and should instead favour allowing smaller gatherings with known contacts.

## Competing interests

No relevant disclosures.

## Supporting information

Survey text and supplementary results
