# Supplementary material for: Modelling the impact of relaxing COVID‐19 control measures during a period of low viral transmission
Source: Med J Aust. 2020 Nov 18;214(2):79–83. doi: 10.5694/mja2.50845 (PMC7753668; doi:10.5694/mja2.50845)
Supplement: Supplementary file 1 — Survey text and supplementary results [file MJA2-214-79-s001.pdf]

The Medical Journal of Australia

Supporting Information

Survey methods and results

**This appendix was part of the submitted manuscript and has been peer reviewed.
It is posted as supplied by the authors.**

Appendix to: Scott N, Palmer A, Delport D, et al. Modelling the impact of relaxing COVID-19 control measures during a period of low viral transmission. *Med J Aust* 2021; doi: 10.5694/mja2.50845.

A. Model description

Contact networks

The model allows people to be a part of multiple independent contact networks. Within each network, a "contact" is a link between two people indicating that transmission would be possible if one of them were infected. The model is designed so that each individual can be a part of an arbitrary number of contact networks used to approximate transmission dynamics associated with different activities or specific public spaces. For this analysis, we considered networks and settings most likely to be subject to a policy change in Australia, with contact networks explicitly modelled for: households; schools; workplaces; social networks; cafés and restaurants; pubs and bars; public transport; places of worship; professional sport; community sport; beaches; entertainment (cinemas, performing arts venues etc); national parks; public parks; large events (concerts, festivals, sports games etc.); child care; and aged care.

Each contact network is defined by a set of properties: the percentage (and age range) of the population who are a part of it; the mean number of contacts per day associated with these activities; whether the contacts are known or random; the type of network structure (random or cluster; for example, public transport is random while schools/workplaces are clustered); the risk of transmission relative to a household contact (scaled to account for frequency of some activities); the effectiveness of contact tracing that might occur; and the effectiveness of quarantine at reducing transmission (for example, quarantine may be effective for workplace transmission, not effective for household transmission, and partially effective for community transmission due to imperfect adherence).

Details of the contact networks are provided in Supporting Information, D.

Model initialisation: household size and age structure

The model population was initialised through the generation of households. Individual households were explicitly modelled based on the household size distribution for Australia [1], with each person in the model assigned to a house. To assign people in the model an age, a single adult was selected for each household as an index, whose age was randomly sampled from a subset of the Victorian adult population (all adults 22 years and older and a percentage of 18-21 year olds - 20%, 40%, 60%, and 80% of people aged 18, 19, 20 and 21, respectively) to ensure that at least one adult was in each household. The age of additional household members was then assigned according to Australian age-specific household contact estimates (from Prem et al. [2], Figure 2), by drawing the age of the remaining members from a probability distribution based on the row corresponding to the age of the index member. The resulting age distribution of the model population, compared to the Victorian population, is provided in Figure 1.

Other contact networks

School classrooms were explicitly modelled. Classroom sizes were drawn randomly from a Poisson distribution with mean 21, the Victorian mean [3]. People in the model aged 5-18 years were assigned to classrooms with people of the same age. Each classroom had one randomly selected adult (>21 years) assigned to it as a teacher. The school contact network was then created as a collection of disjoint, completely connected clusters (i.e. classrooms).

Similarly, a work contact network was created as a collection of disjoint, completely connected clusters of people aged 18-65 years. The size of each cluster was drawn randomly from a Poisson

distribution with mean equal to the estimated mean number of daily work contacts (Table 4). Other clustered contact networks, such as places of worship, community sports, professional sports, child care and aged care were generated analogously (with transmissibility scaled to account for event frequency; Supporting Information, D).

Random contact networks (e.g. public transport) were generated by allocating each person a number of contacts drawn from a Poisson distribution with mean as per Supporting Information, D. Unlike the clustered contact networks, the contacts in random contact networks were resampled at each time step in the model (representing days).

Modelling interventions and policy changes

Policy scenarios modelled were informed by the COVID-19 public health response in Victoria [4] and the *COVIDSAFE Australia* framework [5], and included scenarios related to: the effectiveness of contact tracing; compliance with physical distancing; restricting access to hospitality and entertainment venues and other public spaces; restricting access to places of worship; restricting the size of social gathering; restricting community and professional sport; closing schools and childcare settings; closing non-essential workplaces, retail outlets and health care; and restricted travel across jurisdictional borders and domestic travel.

Each policy change is linked to one or more networks, and can potentially influence the whole population. For example, if non-essential work begins, this would increase the size of the work network, as well as increasing transmissibility in public transport. See Supporting Information, E for full list of modelled scenarios.

Model parameters

Epidemiological data for the daily number of tests conducted, new diagnoses and new severe cases, critical cases and deaths was obtained from the Victorian Department of Health [6, 7]. Newly diagnosed cases were classified as "imported" to Victoria if their mode of acquisition was listed as travel overseas.

Disease specific parameters, including duration of incubation, infectious and symptomatic periods, and age-specific risks associated with disease severity and outcomes, were based on global published estimates (Tables 1 and 2).

Parameters for contact networks and the effect of policy changes were obtained from a combination of the literature and a modified Delphi process (Supporting Information, D). The modified Delphi process involved creation of a panel of 12 experts (a mixture of modellers, epidemiologists, qualitative researchers, social network researchers, infectious disease physicians and public health physicians), who participated in a video conference where they were introduced to the model and the interpretation of parameters. Panel members were then asked to make independent estimates of unknown parameters, which were collated and de-identified by the study team, and the median and range of each parameter was extracted. A follow-up video conference was held where the panel discussed the results and uncertainties and were provided an opportunity to revise any estimates. The distribution of responses for each parameter, as well as the final parameters used, are provided in Supporting Information, part D.

B. Age and network structure of the model

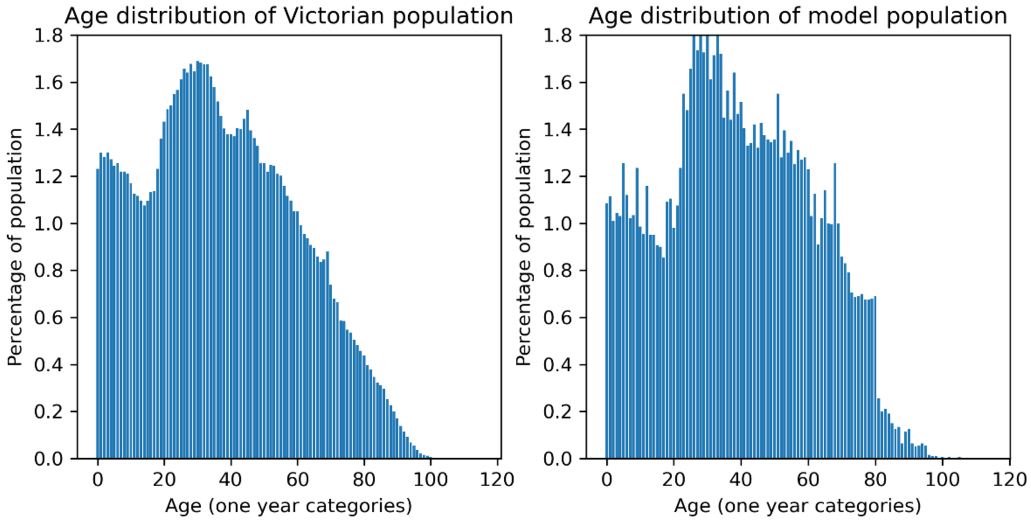

Figure 1. Age distribution (input *v* modelled).

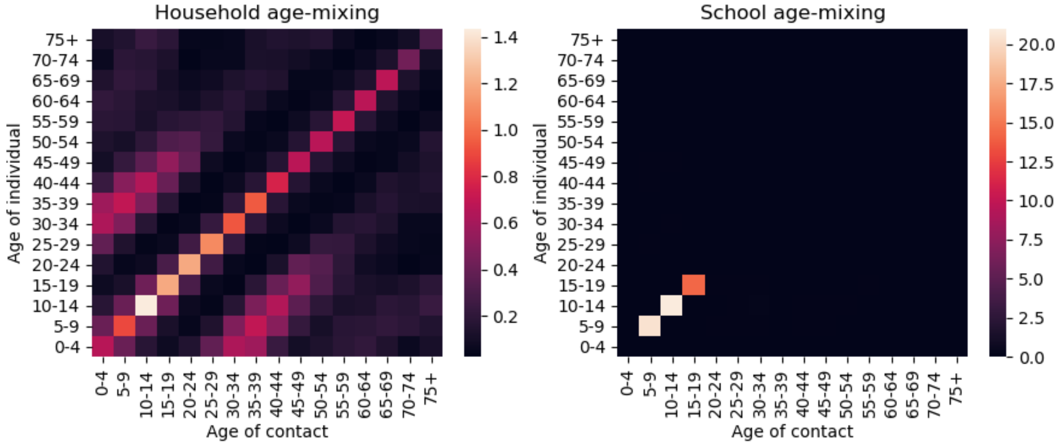

Figure 2. Age mixing within households and schools. The y-axis represents the age of the individual and the x-axis represents the age of their contacts. The colour represents the population-mean number of daily contacts with people of each age. Left: household mixing, based on estimates from Prem et al. [2]. Right: within schools, students aged 5-18 were in classrooms with a mean of 21 students (of the same age) and one teacher. Note that the mean number of contacts for the 15-19 age bracket is slightly lower as 19 year olds do not attend school; and also that contacts between students and teachers and between teachers and students are not visible due to the scale.

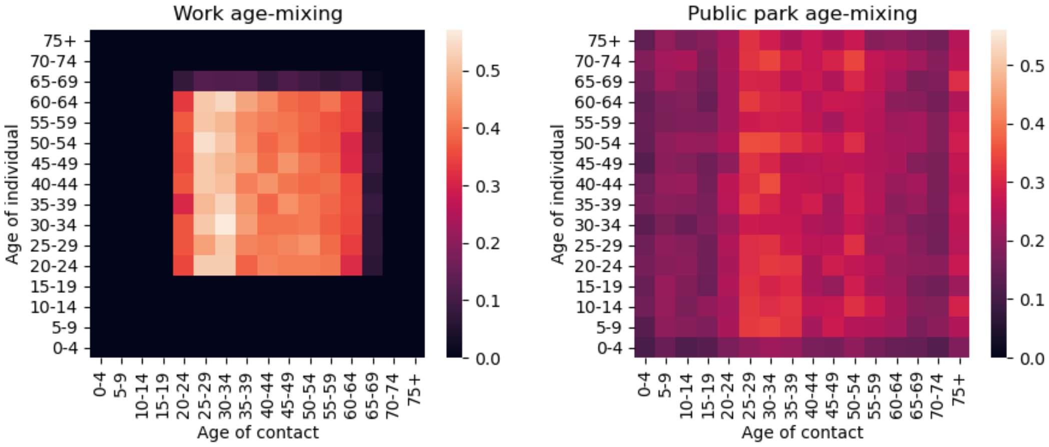

Figure 3. Examples of age-mixing within workplaces and public spaces. The y-axis represents the age of the individual and the x-axis represents the age of their contacts. The colour represents the population-mean number of daily contacts with people of each age. Left: at workplaces, adults aged 18-65 could mix with adults of any other age. The higher mean number of contacts with people aged 25-35 (brighter vertical bands) is due to the disproportionate population age distribution in Victoria (Figure 1). Right: in public spaces, all ages could mix together. Again, the higher mean number of contacts of ages 25-35 is due to the disproportionate population age distribution in Victoria; and the slightly higher mean number of contacts with the 75+ age bracket is because more it covers a greater age range.

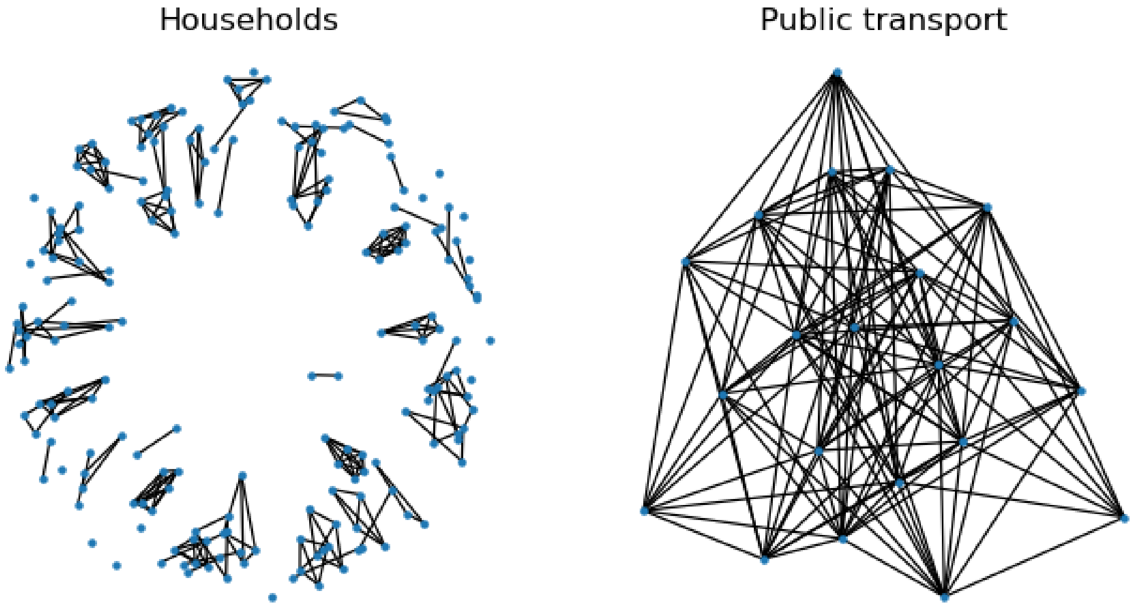

Figure 4. Example contact network structures between in the model. Left: the household network was modelled based on Australian household size distribution data, and was fixed throughout a simulation. Right: some the community transmission networks, such as public transport, were modelled such that each individual had a number of contacts that were randomly assigned, and were re-assigned each day.

C. Model parameters

Table 1. Model parameters

Description	Value	Source
Disease-related parameters	Distribution (mean, std)	
Period from exposure to infectiousness	Lognormal(4.6,4.8)	From Lauer et al., 2020 [8]; additional sources: Du et al., 2020 [9]; Nishiura et al., 2020 [10]; Pung et al., 2020 [11]
Period from infectious to symptomatic	Lognormal(1,1)	He et al., 2020 [12] report that infectiousness started from 2.3 days (95% CI, 0.8–3.0 days) before symptom onset and peaked at 0.7 days (95% CI, −0.2–2.0 days) before symptom onset. Gatto et al., 2020 [13] estimate a pre-symptomatic period of 1.3 days.
Duration for asymptomatics to recover	Lognormal(8,2)	Wolfel et al., 2020 [14]
Duration for mild symptoms to recover	Lognormal(8,2)	Wolfel et al. [14]
Duration for severe symptoms to recover	Lognormal(14,2.4)	Verity et al. [15]
Duration for critical symptoms to recover	Lognormal(14,2.4)	Verity et al. [15]
Duration for critical symptoms to death	[mean=5.1 days, std=1.7 days]	Verity et al. [15]
Other model assumptions		
Transmission rate	Calibrated parameter to fit epidemic data	
Relative change in transmission risk when asymptomatic	0.5	Assumption
Proportion undiagnosed in initial epidemic wave	40%	Assumption
Future testing numbers	10,000 per day	Assumption based on recent testing blitz in Victoria
Sensitivity of test	70%	Expert opinion
Days between having a test and getting result	1 day	Based on current turnaround time for tests
Relative probability of symptomatic people being tested, compared to others	100	Assumption based on symptomatic testing policies

Table 2. Age-specific susceptibility, disease progression and mortality risks

	0-9	10-19	20-29	30-39	40-49	50-59	60-69	70-79	80+	Sources
Relative susceptibility	0.34	0.67	1.00	1.00	1.00	1.00	1.00	1.24	1.47	Zhang et al. [16]
Prob[symptomatic]	0.50	0.55	0.60	0.65	0.70	0.75	0.80	0.85	0.90	Assumed
Prob[severe]	0.00004	0.00040	0.01100	0.03400	0.04300	0.08200	0.11800	0.16600	0.18400	Verity et al. [15]; CDC [17].
Prob[critical]	0.0004	0.00011	0.0005	0.00123	0.00214	0.008	0.0275	0.06	0.10333	CDC [17]
Prob[death]	0.00002	0.00006	0.00030	0.00080	0.00150	0.00600	0.02200	0.05100	0.09300	Verity et al. [15]; Ferguson et al. [18]; CDC [17]

D. Behavioural and contact network parameters for Victoria

The parameters in this appendix were obtained from the literature where available, or through a modified Delphi process where studies were not available (a Delphi process modified to be possible during the COVID-19 pandemic). The Delphi method involves the creation of a group of experts, who anonymously reply to surveys and then receive feedback in the form of a statistical representation of the “group response”. After seeing the group response, the process repeats itself and the group of experts are provided an opportunity to amend their responses, with the goal of subsequent iterations to reduce the range of responses and achieve an approximate expert consensus. The Delphi method is a widely accepted estimation technique, which has been applied across a number of areas of health and social science [19, 20].

For this study, a group of 12 experts (a mixture of modellers, epidemiologists, qualitative researchers, social network researchers and public health and infectious disease clinicians) were invited to participate. A video conference was held where they were introduced to the model and the interpretation of parameters, and participants were asked to make independent estimates of unknown parameters following the conference. Estimates were then collated by the study team, and the median and range of each parameter was extracted. A follow-up video conference was held where the panel discussed the results, uncertainties and were offered an opportunity to update any parameters. In this appendix, the distribution of responses are provided or each model parameter.

Population subsets

Each contact network only applies to a subset of the model population; because not everyone participates in each activity, or attends each location, only a subset are able to be infected at these places or during these activities. The subset of the population that each network applies to is defined as a percentage of a given age range.

Table 3. Population subsets included in each contact network

Contact network associated with	Age group	% of age group	Source/Calculation
General community transmission	all	100%	All individuals are assumed to contribute to general community transmission
Places of worship	all	11%	11% of the population attend church at least weekly [21]
Professional sport	18-40	0.06%	Approximated as just Australian Rules Football (AFL) as an illustrative example. Estimated 1,800 people involved in AFL divided by approximately 3 million Victorians.
Community sport	4-30	34%	For people under 30, age-weighted participation rate of 34%. Over 30 years ignored as rates quickly decline [22].
Beaches	0-80	15%	Median estimate from panel:<br>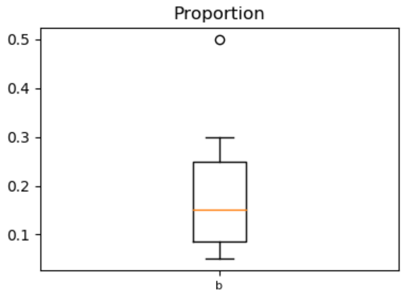

Contact network associated with	Age group	% of age group	Source/Calculation
Entertainment (cinemas, performing arts venues etc)	15+	40%	Median estimate from panel: 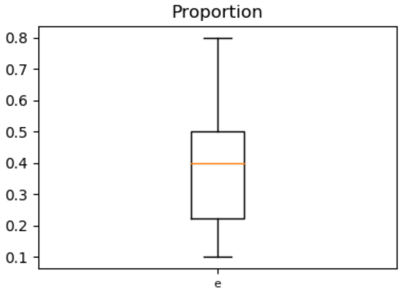
Cafés and restaurants	18+	60%	Participation by age groups <18 considered to be small rather than 18+.<br>Percentage of age group based on median estimates of panel: 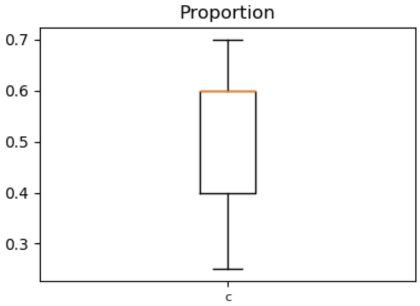
Pubs and bars	18+	40%	Median estimate from panel: 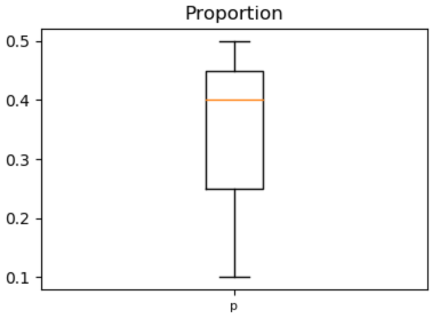
Public transport	15+	11.5%	2016 census. 11.5% of people travelled to work by public transport [23].
National parks	all	5.6%	1.38 million national park visitors in Australia in 2017 [24], with an Australian population size of 24.6 million.<br>"national park goers" are over counted due to multiple visits, however conversely this estimate does not include state parks. This would give ~5.6% (1.38 million / 24.6 million).
Public parks	all	60%	Median estimate from panel: 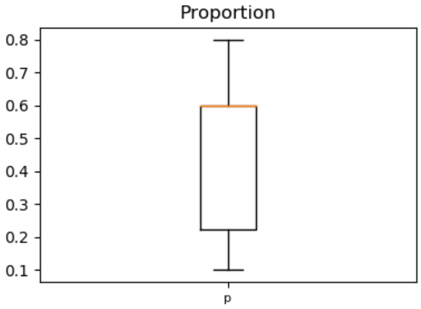
Large events (concerts,	all	15%	Median estimate from panel:

Contact network associated with	Age group	% of age group	Source/Calculation
festivals, sports games etc.)			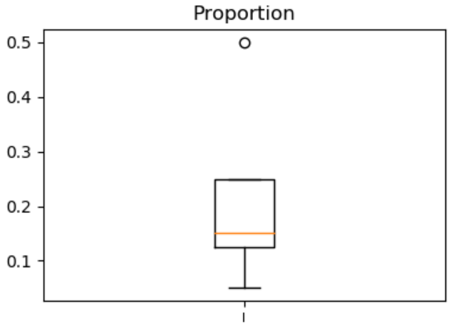
Child care	1-6	54.5%	~54.5% of children were in some form of childcare [25]
Social networks	15+	100%	Assumed entire population has social network
Aged care	65+	7%	7% of Australians 65+ accessed residential aged-care [26].

Network structure and size

Each network can have a different structure, with people either being connected to their contacts randomly ("random") or people being grouped into disconnected clusters ("clustered", e.g. schools, where the network consists of disjoint classrooms, with students in each classroom connected to one another). The differences between a random and clustered network are illustrated in Figure 4.

Each person in the model has a specified number of contacts in each network layer. The epidemiological definition of a contact between two people is used, where a contact is defined as having a 15-minute face-to-face conversation, or spending one hour or more in a room together. For those who have a non-zero number of contacts in a particular network (i.e. they are inside the applicable age range and randomly-selected population fraction defined in Table 3), if the contact network is "random" type, then their number of contacts is drawn from a Poisson distribution with mean as per Table 4. If the contact network is "clustered", then the size of each cluster is drawn from a Poisson distribution with mean as per Table 4.

Networks can also be time-varying or not. For example, contact networks for public spaces (e.g. public transport) are regenerated each day, to simulate once-off mixing, compared to work networks in which specific individuals remain connected to one another.

Table 4. Model inputs for the mean number of contacts per person in settings or during activities

Parameter	Network type	Time-varying contacts?	Contacts per day (when participating in event)	Source/Calculation
Schools	Clustered	No	21	Mean classroom size in Victoria [27]
Work	Clustered	No	5	Age-weighted Australian estimates from Prem et al. [2]
Community	Random	Yes	1	Minimal amount, to cover other forms of transmission not being modelled.
Places of worship	Clustered	No	20	Median estimate from panel: 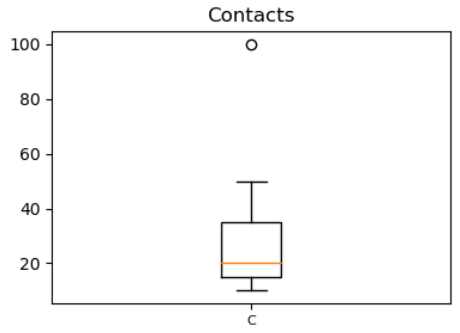

Parameter	Network type	Time-varying contacts?	Contacts per day (when participating in event)	Source/Calculation
Professional sport	Clustered	No	40	Median of estimate from panel:<br>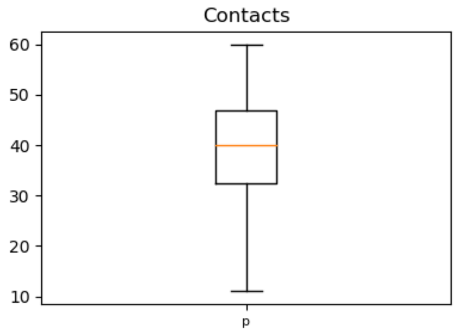
Community sport	Clustered	No	30	Median of estimate from panel:<br>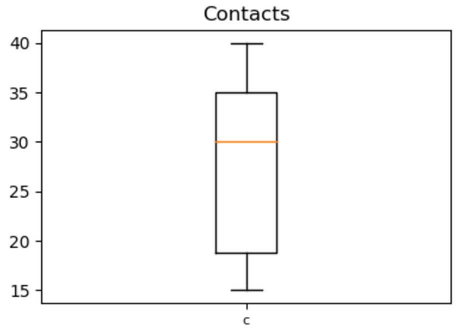
Beaches	Random	Yes	8	Median estimate from panel:<br>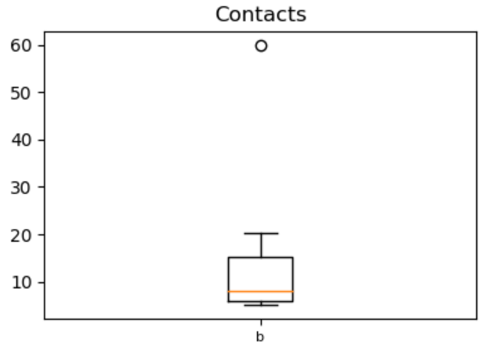
Entertainment (cinemas, performing arts venues etc)	Random	Yes	25	Median of estimate from panel:<br>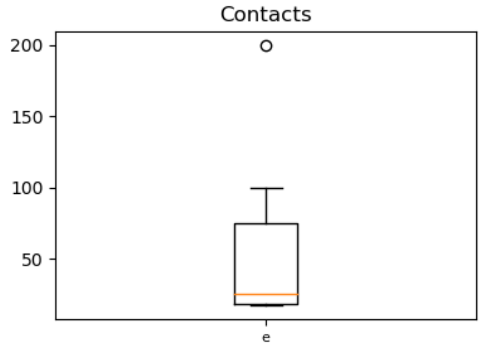

Parameter	Network type	Time-varying contacts?	Contacts per day (when participating in event)	Source/Calculation
Cafés and restaurants	Random	Yes	19	Median of estimate from panel:<br>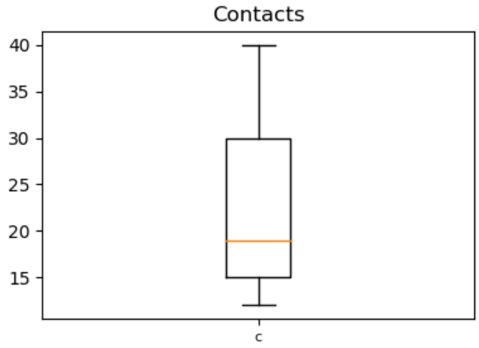
Pubs and bars	Random	Yes	30	Median of estimate from panel:<br>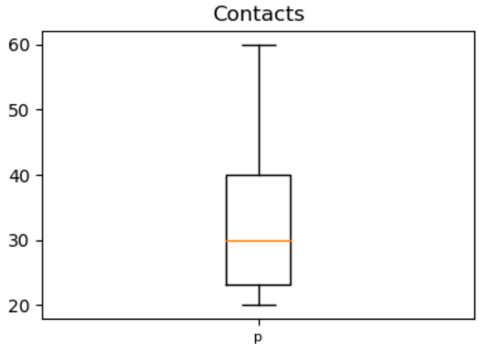
Public transport	Random	Yes	25	Median estimate from panel:<br>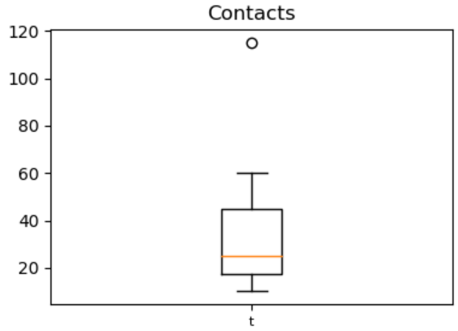
National parks	Random	Yes	6	Median estimate from panel:<br>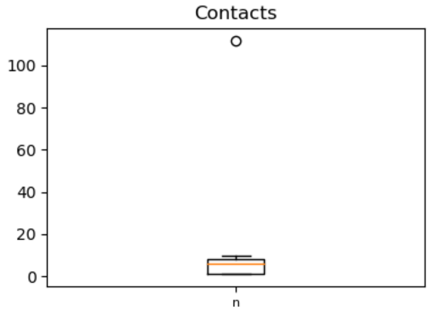

Parameter	Network type	Time-varying contacts?	Contacts per day (when participating in event)	Source/Calculation
Public parks	Random	Yes	10	Median of estimate from panel:<br>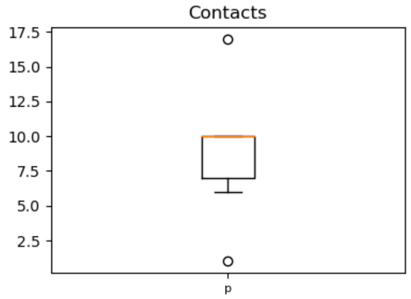
Large events (concerts, festivals, sports games etc.)	Random	Yes	50*	Median estimate from panel:<br>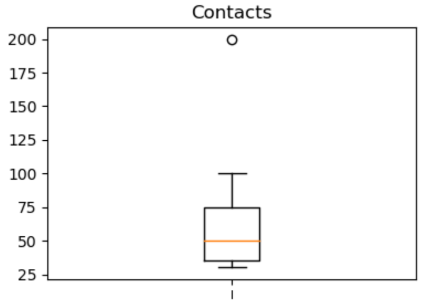
Child care	Clustered	No	20	Median estimate from panel:<br>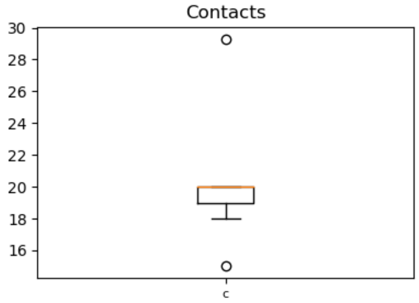
Social networks	Random	No	6	Median estimate from panel:<br>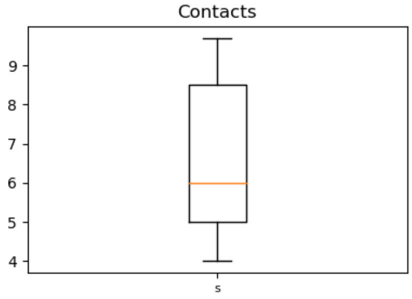

Parameter	Network type	Time-varying contacts?	Contacts per day (when participating in event)	Source/Calculation
Aged care	Clustered	No	12	Median estimate from panel: 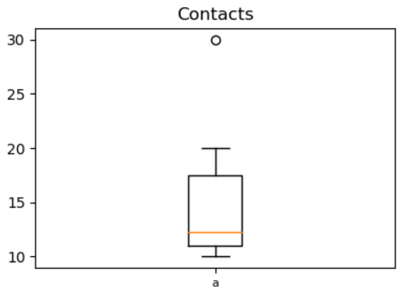

*Not size of large event but number of actual contacts during event

Relative transmissibility of contact networks

Transmission of COVID-19 is likely to be highly variable depending on network. As well as an overall daily risk of transmission per contact (the calibration parameter for the model), the risk of transmission per contact per day is different for each network. Table 5 shows these estimated differences relative to the transmission risk per contact per day within households.

Table 5: Relative risk of transmission through a contact, compared to a household contact. No studies were available for these parameters, meaning that they were all are based on the median of the expert panel's estimates shown in Figure 9 below.

Parameter	Relative transmission risk (compared to household)
Households	*1.0 (reference)*
Schools	0.50
Work	0.50
Community	0.10
Places of worship	0.30
Professional sport	0.70
Community sport	0.50
Beaches	0.10
Entertainment (cinemas, performing arts venues etc)	0.20
Cafés and restaurants	0.30
Pubs and bars	0.40
Public transport	0.30
National parks	0.10
Public parks	0.20
Large events (concerts, festivals, sports games etc.)	0.25
Child care	0.50
Social networks	0.45
Aged care	0.80

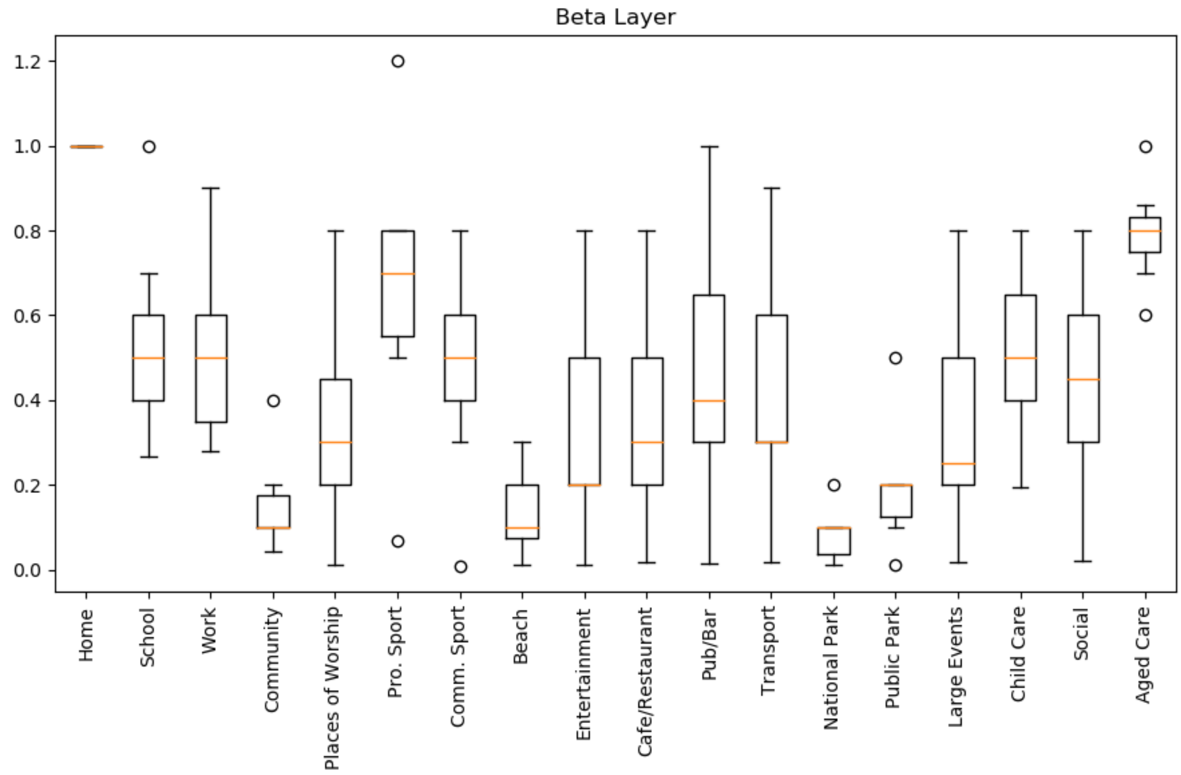

Figure 5. Expert panel estimates for the risk of transmission in each contact network, relative to household contacts

Event frequency

People may not typically interact with the activities and public spaces corresponding to each network on a daily frequency; for example, community sport might be played once per week. The model currently does not include simulation of each activity with different frequencies, and so the impact of this was approximated by reducing the relative transmission risk in each contact network.

The relative transmissibility (Table 5) was divided by the activity frequencies/365 to develop a proxy for per-day transmission risk.

Table 6. Event frequency

Parameter	Mean number of days in year	Source/calculation
Work	206	Calculated from ABS data [28]. Monthly hours worked/employed persons gives mean monthly hours worked. Then assumed that working day is 8 hours, giving a mean of 17.14 days worked per month
Community	365	General community transmission assumed to occur everyday
Places of worship	52	One service per week
Professional sport	100	Median event frequency estimate from panel: 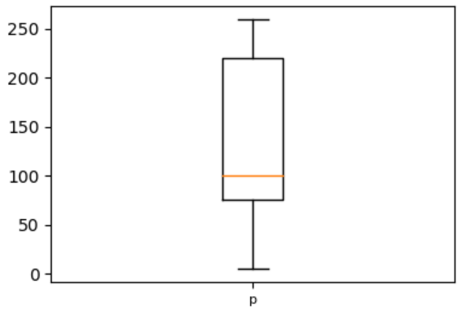
Community sport	52	Median event frequency estimate from panel: 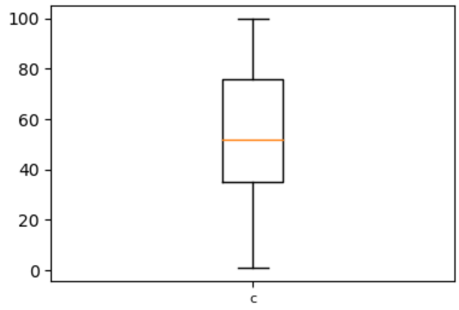

Parameter	Mean number of days in year	Source/calculation
Beaches	26	Median event frequency estimate from panel: 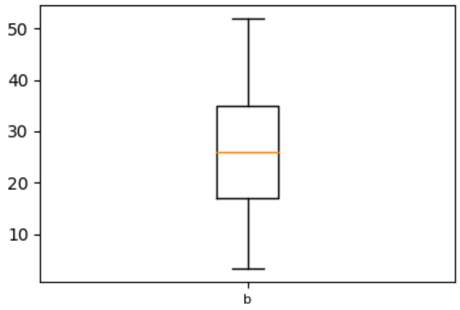
Entertainment (cinemas, performing arts venues etc)	15	Median event frequency estimate from panel: 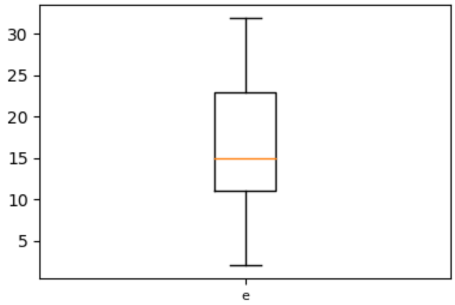
Cafés and restaurants	52	Median event frequency estimate from panel: 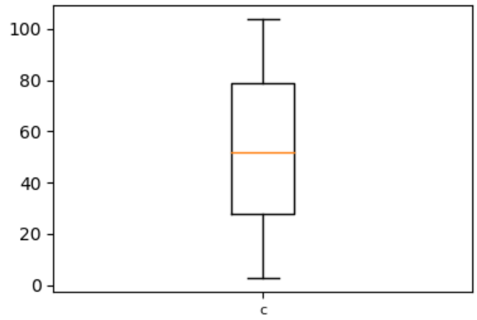
Pubs and bars	52	Median event frequency estimate from panel: 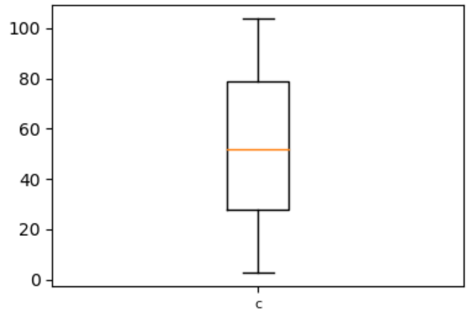

Parameter	Mean number of days in year	Source/calculation
Public transport	200	Median event frequency estimate from panel: 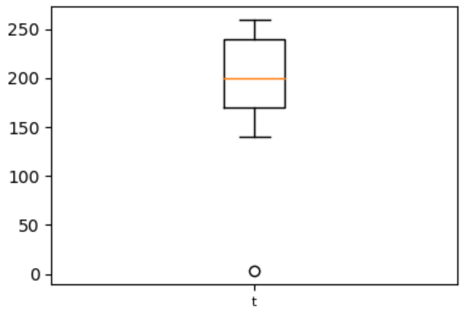
National parks	12	Median event frequency estimate from panel: 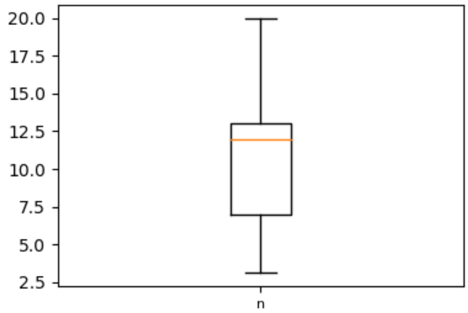
Public parks	52	Median event frequency estimate from panel: 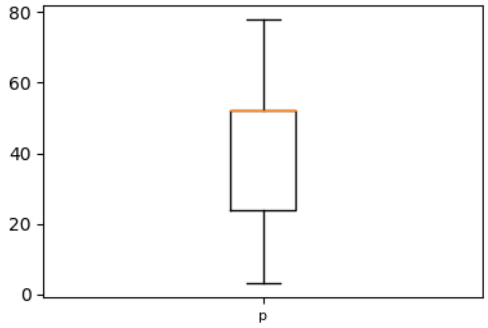
Large events (concerts, festivals, sports games etc.)	10	Median event frequency estimate from panel: 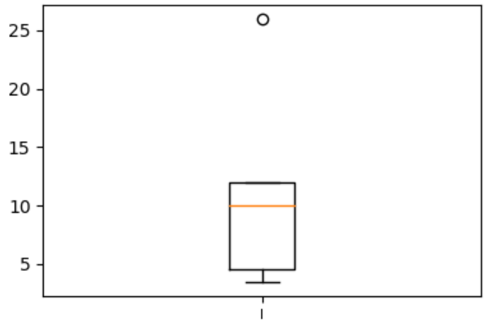

Parameter	Mean number of days in year	Source/calculation
Child care	200	Median event frequency estimate from panel: 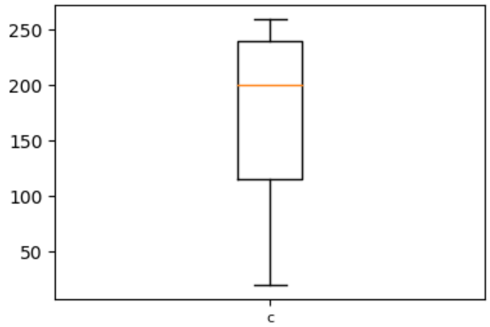
Social networks	52	Median event frequency estimate from panel: 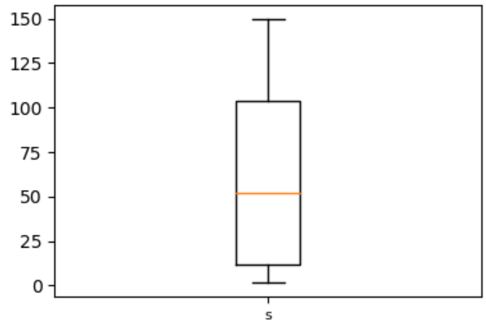
Aged care	365	Residents assumed to be in fulltime care

Quarantine and contact tracing

People who are asked to self-isolate are likely to change their behaviour in ways that reduce their likelihood of transmission through different contact networks. For people in quarantine, their relative transmissibility in each contact network (Table 5) is reduced by the factors shown in Table 7. For example, quarantine is modelled to have no impact on household transmission, to completely stop workplace and school transmission, and reduce (but not stop) other forms of community transmission due to imperfect adherence.

When a person is diagnosed, there is a probability of tracing the people they are connected to in different contact networks, and an associated time to trace them. For example, we assume that household members would be notified on the day of diagnosis, while workplace contacts would have a 70% chance of being traced within 2 days.

The effectiveness of quarantine, contact tracing probabilities and tracing time were estimated from the expert panel.

Table 7. Effectiveness of quarantine and contact tracing on different contact networks. No studies were available for these parameters, meaning that they were all are based on the median of the expert panel's estimates.

Parameter	Quarantine effectiveness	Probability of successful contact tracing	Time to trace contact
Households	1.00	1.00	1
Schools	0.01	0.95	2
Work	0.10	0.80	2
Community	0.20	0	N/A
Places of worship	0.01	0.50	5
Professional sport	0.00	0.80	3
Community sport	0.00	0.50	3
Beaches	0.00	0	N/A
Entertainment (cinemas, performing arts venues etc)	0.00	0	N/A
Cafés and restaurants	0.00	0	N/A
Pubs and bars	0.00	0	N/A
Public transport	0.01	0	N/A
National parks	0.00	0	N/A
Public parks	0.00	0	N/A
Large events (concerts, festivals, sports games etc.)	0.00	0	N/A
Child care	0.01	0.95	2
Social networks	0.00	0.90	3
Aged care	0.20	0.95	2

Intervention effectiveness

There were no studies available to estimate the impact of policy changes on each network. However, for many polices, the impact is based on turning on / off transmission within a particular network, and so the impact is derived from the network properties in Tables 3-6.

For some policies, there are logical impacts that extend beyond their specific network; for example, if non-essential work is cancelled, then the transmission risk on public transport would be expected to decrease. For these auxiliary effects, the actual impact size is unknown, and so has been estimated by the panel of experts.

Table 8. Impact of policies. Data were not available to inform changes in transmission due to different policies. All estimates are based on median values reported

Description	Parameter changes (compared to pre-COVID time)
Physical distancing communication and enforcement	86% decrease in overall beta*
Physical distancing communication and enforcement relaxed a bit (when restrictions begin to be lifted)	When physical distancing is relaxed, overall hygiene and physical distancing benefits are reduced by 75% (from 86% reduction (see above) to only a 35% reduction)
Beaches closed	0 transmission risk in beach network
Beaches restricted to groups of 2	80% decrease in transmission risk within beach network
Beaches restricted to groups of <10	40% decrease in transmission risk within beach network
National and state parks closed	0 transmission risk in national park network
Places of worship closed	0 transmission risk in places of worship network
Places of worship implementing 4 sq m rule	40% decrease in transmission risk within places of worship network
Cafes and restaurants take-away only	• 10% increase in transmission risk at home<br>• 0 transmission risk in café_restaurant network
Cafes and restaurants implementing 4 sq m physical distancing rule	50% decrease in transmission risk within café_restaurant network
Pubs and bars closed	• 10% increase in transmission risk at home<br>• 0 transmission risk in pub_bar network
Pubs and bars implementing 4 sq m physical distancing rule	40% decrease in transmission risk within pub_bar network
Outdoor settings restricted to <2 people	• 20% increase in transmission risk at home<br>• 30% decrease in general community transmission risk<br>• 30% decrease in transmission risk in transport network<br>• 0 transmission risk in entertainment network<br>• 0 transmission risk in national park network<br>• 60% decrease in transmission risk in public park network<br>• 0 transmission risk in large event network<br>• 70% decrease in transmission risk in social networks

Description	Parameter changes (compared to pre-COVID time)
Outdoor settings restricted to <10 people	• 5% increase in transmission risk at home<br>• 20% decrease in transmission risk in general community network<br>• 0 transmission risk in entertainment network<br>• 30% decrease in transmission risk in transport network<br>• 30% decrease in in transmission risk in public park network<br>• 0 transmission risk in large event network
Outdoor settings restricted to <200 people	• 20% decrease in transmission risk in transport network<br>• 0 transmission risk in large event network
Professional sports cancelled for players (crowds are different policy)	0 transmission risk in pSport network
Community sports cancelled	0 transmission risk in cSport network
Child care closed	0 transmission risk in child_care network
Schools closed	• 50% decrease in transmission risk in school network<br>• 90% of children removed from school network
Non-essential retail outlets, including shopping centres closed	• 30% decrease in transmission risk in general community network<br>• 5% of workers are removed from work network
Cinemas, performing arts venues etc. closed	0 transmission risk in entertainment network
concerts, festivals, sports games etc.	0 transmission risk in large event network
Non-essential work closed	• 33% reduction in transmission risk on public transport<br>• 20% of workers are removed from work network
Non-COVID-19 health services closed	5% of workers are removed from work network
Travel across state borders allowed and increased domestic travel	imported infections increases to 5 per day
social catch ups with <10 people banned	0 transmission risk in social network
Enhanced screening and distancing within age care facilities	0 transmission risk in aged care network

* From Flutracker, 0.2% fever and cough prevalence compared to ~1.4% the same time last year --> 86% reduction [29].

E. Policy changes to be simulated in the model

Interventions can be modelled by changing parameters dynamically throughout a simulation. At any time point in a simulation, parameters can be varied to:

- Change the number of imported infections (from other Australian jurisdictions or internationally)
- Change the number of tests per day
- Change adherence to quarantine after diagnosis
- Scale the overall probability of transmission per contact (e.g. due to general hand hygiene)
- Scale the relative transmission risk for specific contact layers (e.g. a policy closing cafes and restaurants would set the transmission risk for the cafe/restaurant network to be zero)
- Remove a proportion of people from a network (e.g. a policy stopping non-essential work would remove some people from the work contact network)
- Change the effectiveness of contact tracing for a particular contact network (e.g. the COVIDSafe app makes contact tracing possible for community transmission only if both the infected and susceptible person have the app)

Policy changes are linked to one or more networks, and can potentially influence the whole population. For example, if non-essential work begins, this would increase the size of the work network, as well as increasing transmissibility in public transport.

Policy scenarios modelled were informed by the COVID-19 public health response and the COVIDSAFE Australia framework [5]. The following are examples of policies that can be simulated:

1. Contact tracing (including the use of COVIDSafe app for different coverages)
2. Communication and enforcement of physical distancing (e.g. signs, advertisements, policing)
3. Cafes and restaurants take-away only
4. Cafes and restaurants implementing 4 square metre rule physical distancing rule
5. Pubs and bars closed
6. Pubs and bars implementing 4 square metre rule physical distancing rule
7. Places of worship closed
8. Places of worship implementing 4 square metre rule physical distancing rule
9. Outdoor settings restricted to <2 people
10. Outdoor settings restricted to <10 people
11. Outdoor settings restricted to <200 people
12. Indoor social catch ups with <10 people banned
13. Community sports
14. Professional sports (for players)
15. Child care closed
16. Schools closed
17. Entertainment venues closed (e.g. cinemas, performing arts)
18. Large events cancelled (e.g. concerts, festivals, sports games)
19. Beaches closed
20. Beaches restricted to groups of 2
21. Beaches restricted to groups of <10
22. National and state parks closed
23. Non-essential retail outlets closed
24. Non-essential work closed
25. Non-COVID-19 health services closed
26. Travel restrictions across state borders

Any set of interventions can be run in combination, or staged according to policy change dates.

F. Policy changes in Victoria

Summarised from Victorian Department of Health and Human Services (DHHS) coronavirus updates archive [30]:

- 1 Feb: Travel restrictions from China
- 1 Mar: Travel restrictions from Iran
- 5 Mar: travel restrictions from South Korea
- 11 Mar: travel restrictions from Italy
- 15 Mar: gatherings of more than 500 people cancelled
- 15 Mar: all international travellers must self-isolate for 14 days
- 19 Mar: indoor gatherings limited to 100 people
- 20 Mar: Australia closes borders to all non-residents and non-Australian citizens
- 21 Mar: 4 square metre social distancing rule for people in any enclosed spaces
- 22 Mar: pubs, bars, entertainment venues, cafes, cinemas, restaurants, places of worship closed (or take-away only)
- 29 Mar: public gatherings limited to two people.
- 29 Mar: People over 70 years, people with chronic illness over 60 years, or Indigenous Australians over 50 urged to self-isolate
- 29 Mar: only four reasons to leave home: shopping for essentials; for medical or compassionate needs; exercise in compliance with the public gathering restriction of two people; and for work or education purposes

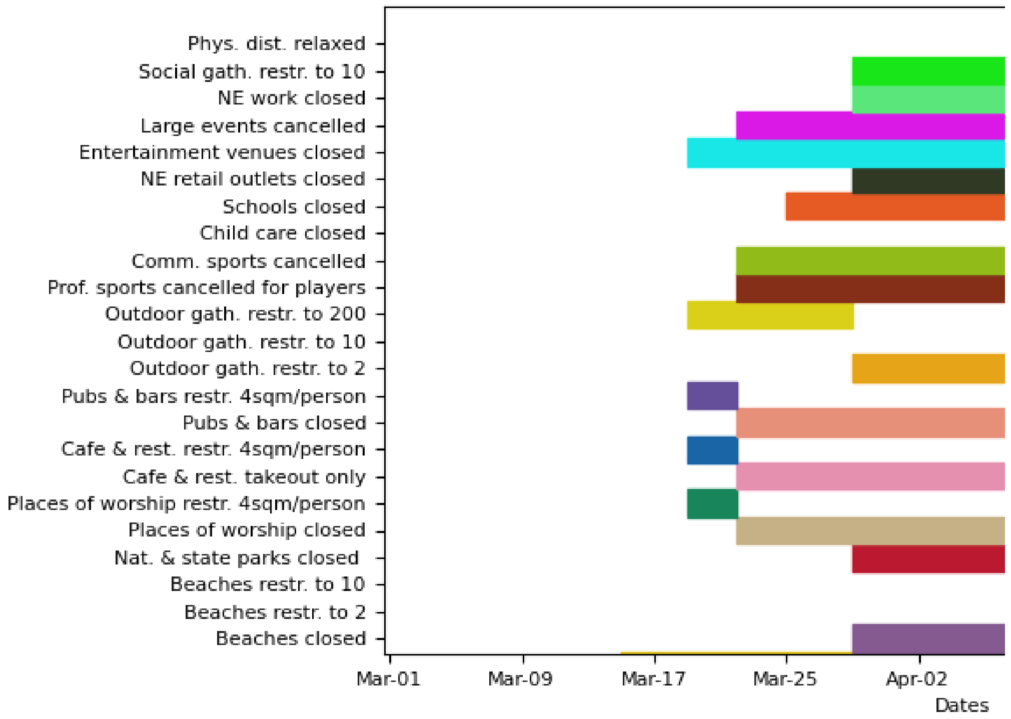

Figure 6. Policy changes and restrictions examined in the model.

G. Supplementary figures

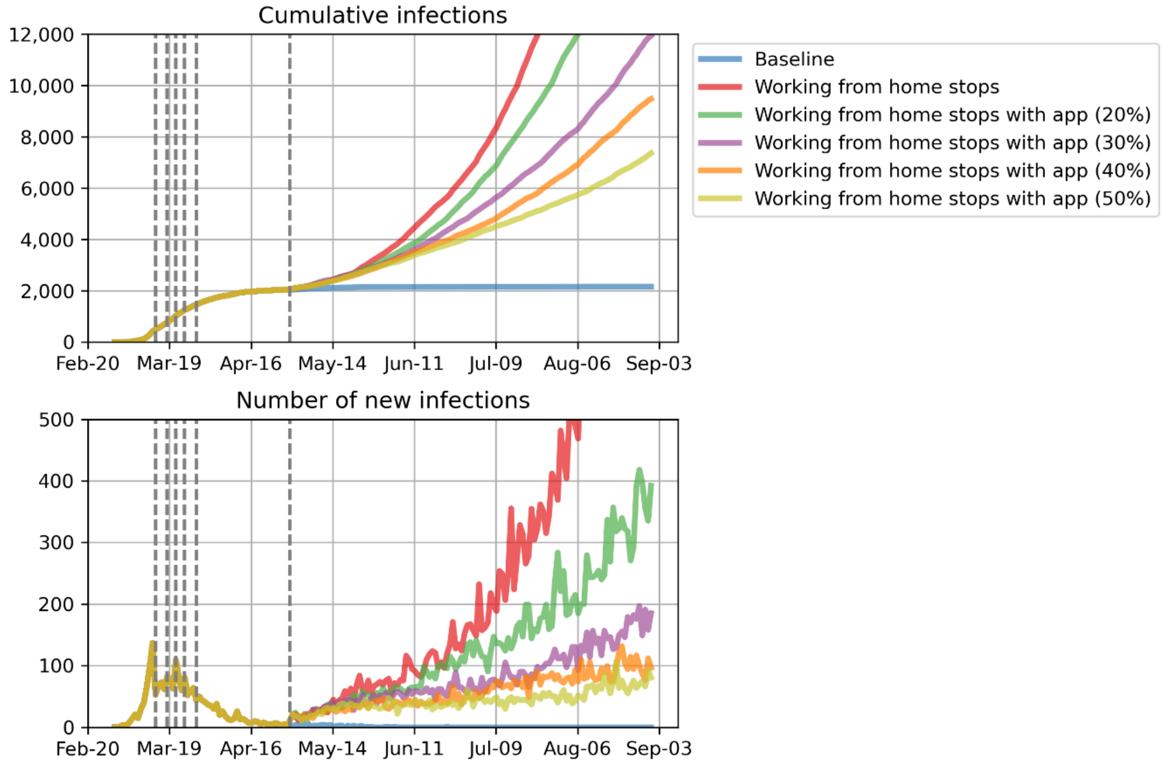

Figure 7. Impact of contact tracing smartphone app. Projected cumulative population-level infections when work from home directives are removed, with different uptake of the smartphone app. Dashed lines show the dates of policy changes.

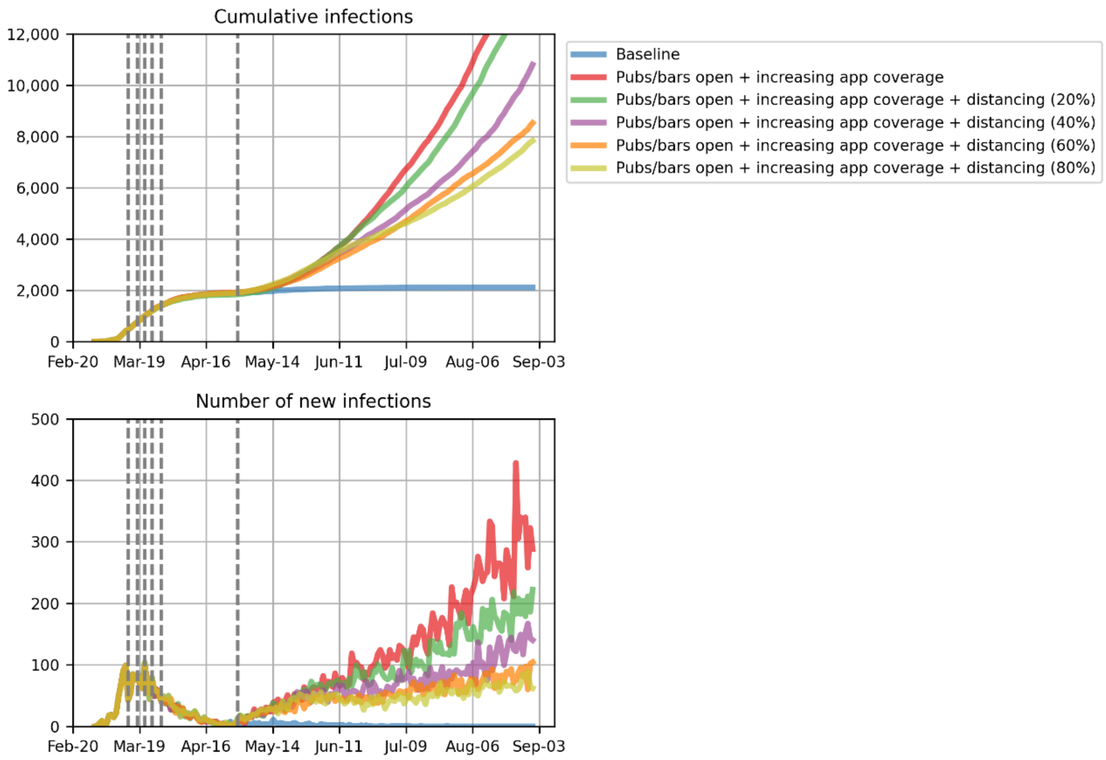

Figure 8. Impact of physical distancing policies in pubs and bars combined with smartphone app coverage scale-up to 25% by 15 June. Projected cumulative population-level infections when pubs and bars are opened, with compulsory identification recording enabling 40-80% of contacts from those venues to be traced within one day of a diagnosed case. Dashed lines show the dates of policy changes.

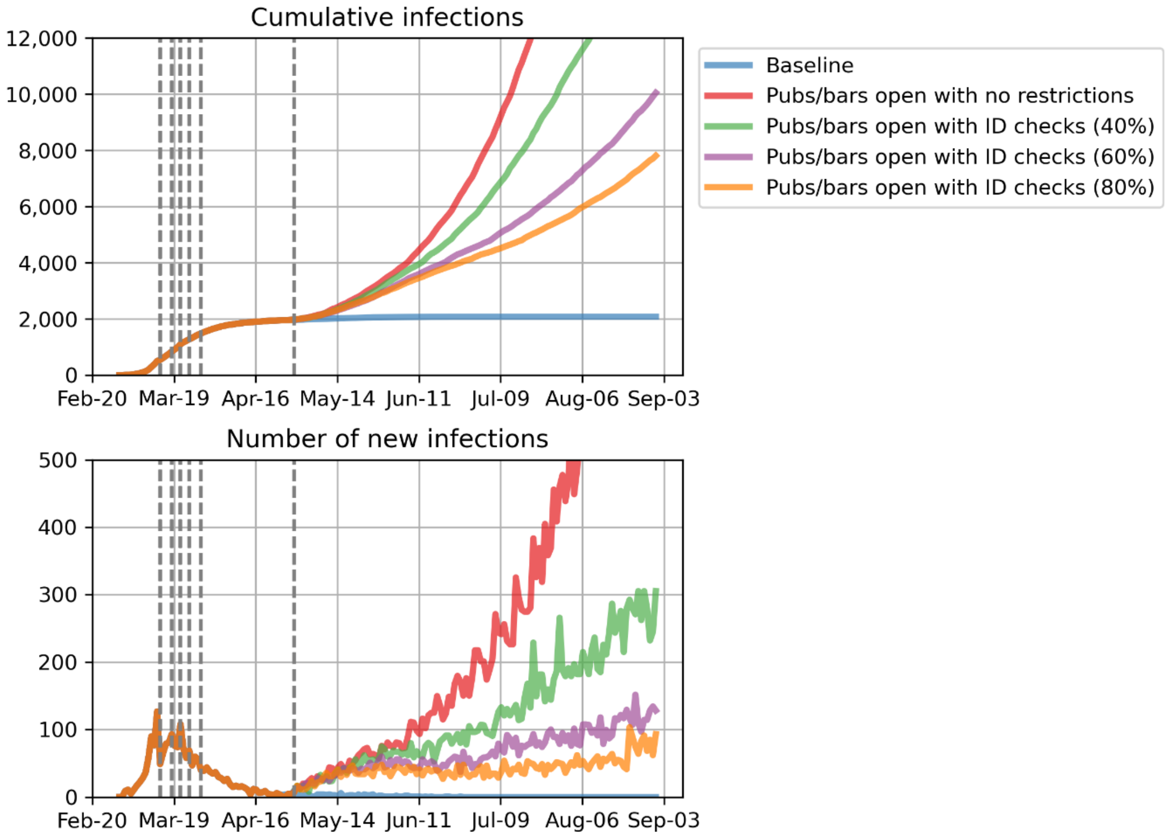

Figure 9. Impact of identification collection alongside the opening of pubs and bars. Projected cumulative population-level infections when pubs and bars are opened, with compulsory identification recording enabling 40-80% of contacts from those venues to be traced within one day of a diagnosed case. Dashed lines show the dates of policy changes. Population-level coverage of the contact tracing smartphone app was set to 5% (estimated coverage at 1 May).

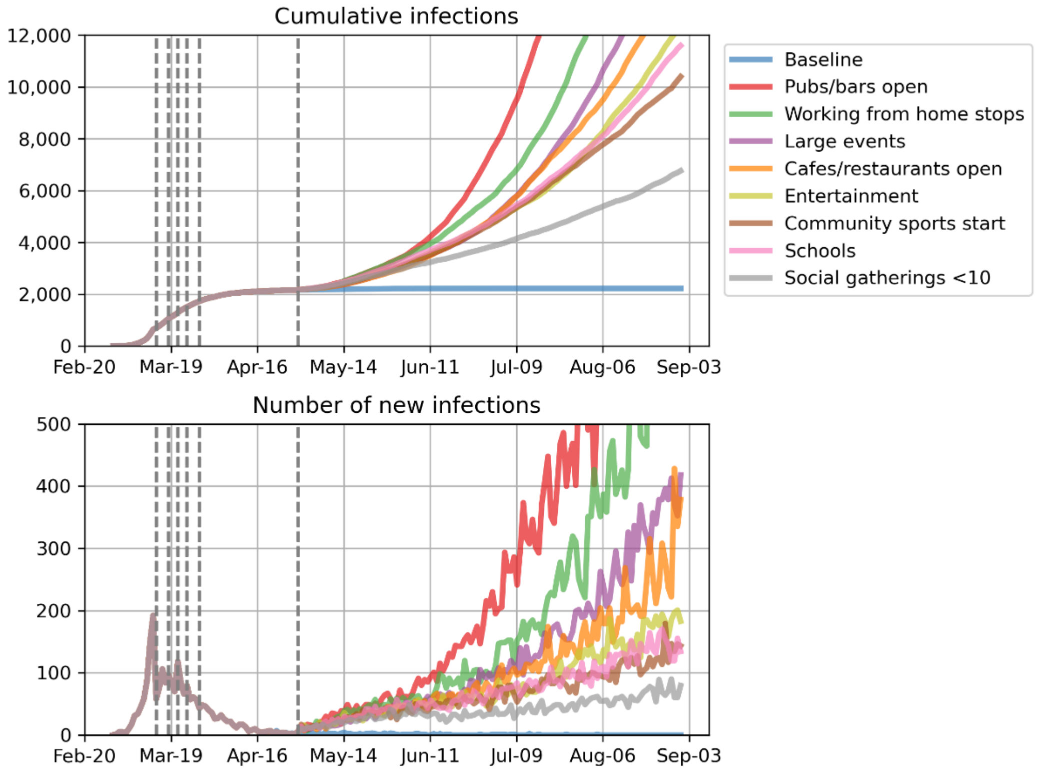

Figure 10. Sensitivity analysis for COVID-19 susceptibility by age. Projected cumulative population-level infections when different policy restrictions are lifted, as per Figure 2, except with people of all ages having equal susceptibility to infection. Note that clinical outcomes were still assumed to vary by age. Compared to baseline estimates, opening schools has slightly worse outcomes, but still minimal compared to other policies due to contacts being known and contact tracing being effective. Minimal impact is seen for scenarios that apply directly to adults (e.g. pubs and bars opening or working from home stopping).

References

1. Idcommunity. Australian household size, 2016 estimates. https://profile.id.com.au/australia/household-size (viewed May 2020).
2. Prem K, Cook AR, Jit M. Projecting social contact matrices in 152 countries using contact surveys and demographic data. *PLoS Comput Biol* 2017; 13: e1005697.
3. Victorian State Government. Victorian school system information. https://www.study.vic.gov.au/en/study-in-victoria/victoria's-school-system/Pages/default.aspx (viewed May 2020).
4. Department of Health and Human Services (DHHS). Victorian COVID-19 restrictions. https://www.dhhs.vic.gov.au/victorias-restriction-levels-covid-19 (viewed May 2020).
5. Australian Department of Health. 3-Step framework for a COVIDSafe Australia. https://www.health.gov.au/resources/publications/3-step-framework-for-a-covidsafe-australia (viewed May 2020).
6. Victorian Department of Health. Power BI Report. https://app.powerbi.com/view?r=eyJrIjoiODBmMmE3NWQtZWNlNC00OWRkLTk1NjYtMjM2YTY1MjI2NzdjIiwidCI6ImMwZTA2MDFmLTBmYWMtNDQ5Yy05Yzg4LWExMDRjNGViOWYyOCJ9 (viewed May 2020)
7. COVID Live. Australian COVID-19 data. https://www.covidlive.com.au (viewed May 2020).
8. Lauer SA, Grantz KH, Bi Q, et al. The incubation period of coronavirus disease 2019 (COVID-19) from publicly reported confirmed cases: estimation and application. *Ann Intern Med* 2020; 172: 557-582.
9. Du Z, Xu X, Wu Y, et al. The serial interval of COVID-19 from publicly reported confirmed cases. *Emerg Infect Dis* 2020; 26: 1341-1343.
10. Nishiura H, Linton NM, Akhmetzhanov AR. Serial interval of novel coronavirus (COVID-19) infections. *Int J Infect Dis* 2020; 93: 284-286.
11. Pung R, Chiew CJ, Young BE, et al. Investigation of three clusters of COVID-19 in Singapore: implications for surveillance and response measures. *Lancet* 2020; 395: 1039-1046.
12. He X, Lau EH, Wu P, et al. Temporal dynamics in viral shedding and transmissibility of COVID-19. *Nat Med* 2020; 26: 672-675.
13. Gatto M, Bertuzzo E, Mari L, et al. Spread and dynamics of the COVID-19 epidemic in Italy: Effects of emergency containment measures. *Proc Natl Acad Sci* 2020; 117: 10484-10491.
14. Wölfel R, Corman VM, Guggemos W, et al. Virological assessment of hospitalized patients with COVID-2019. *Nature* 2020; 581: 465-469.
15. Verity R, Okell LC, Dorigatti I, et al. Estimates of the severity of coronavirus disease 2019: a model-based analysis. *Lancet Infect Dis* 2020; 20: 669-677.
16. Zhang J, Litvinova M, Liang Y, et al. Changes in contact patterns shape the dynamics of the COVID-19 outbreak in China. *Science* 2020; 368: 1481-1486.
17. Centre for Disease Control and Prevention. [COVID-19 Response Team. Severe outcomes among patients with coronavirus disease 2019 (COVID-19)—United States, February 12-March 16, 2020. *MMWR Morb Mortal Wkly Rep* 2020; 69: 343-346.
18. Ferguson N, Laydon D, Nedjati Gilani G, et al.: Report 9: Impact of non-pharmaceutical interventions (NPIs) to reduce COVID19 mortality and healthcare demand. http://hdl.handle.net/10044/1/77482 (viewed May 2020).
19. de Meyrick J. The Delphi method and health research. *Health Educ* 2003; 103: 7-16.
20. Landeta J. Current validity of the Delphi method in social sciences. *Technol Forecast Soc Change* 2006; 73: 467-482.
21. Powell R, Pepper M. Local churches in Australia: research findings from NCLS Research. 2016 NCLS Church Life Pack Seminar Presentation. http://www.2016ncls.org.au/resources/downloads/Local%20Churches%20in%20Australia-Research%20Findings%20from%20NCLS%20Research(2017).pdf (viewed May 2020).

22. The Sport Participation Research Project: sport participation rates, aggregation of 12 sports, Victoria 2017. https://www.vichealth.vic.gov.au/-/media/ResourceCentre/PublicationsandResources/Physical-activity/2017-Sports-Participation-Research-Program.pdf?la=en&hash=CCF0FD75AC59BC45CBD3E1BD62F9EBBE2725D5D9 (viewed May 2020).
23. Australian Bureau of Statistics. 2016 Census estimates on method of travel to work. https://www.abs.gov.au/AUSSTATS/abs@.nsf/mediareleasesbyReleaseDate/7DD5DC715B608612CA2581BF001F8404?OpenDocument (viewed May 2020).
24. Australian Government Director of National Parks. Annual report 2016-17. https://www.environment.gov.au/system/files/resources/1c555a10-dea0-4121-a408-00952eaeae12/files/dnp-annual-report-2016-17-web.pdf (viewed May 2020).
25. Australian Childcare Alliance. Pre-budget submission 2017-18. https://treasury.gov.au/sites/default/files/2019-03/C2016-052_Australian-Childcare-Alliance.pdf (viewed May 2020).
26. Australian Institute of Health and Welfare. Aged care information website. https://www.aihw.gov.au/reports/australias-welfare/aged-care (viewed May 2020).
27. Victorian Department of Education and Training. School classroom sizes. https://www.study.vic.gov.au/en/study-in-victoria/victoria's-school-system/Pages/default.aspx (viewed May 2020).
28. Australian Bureau of Statistics. Labour force estimates (Australia). https://www.abs.gov.au/AUSSTATS/abs@.nsf/Lookup/6202.0Main+Features1Mar%202020?OpenDocument (viewed May 2020).
29. FluTracking. FluTracker weekly report. https://info.flutracking.net/reports-2/australia-reports (viewed May 2020).
30. Victorian Department of Health and Human Services. Coronaviris updates archive. https://www.dhhs.vic.gov.au/coronavirus/updates/202003 (viewed May 2020).
